# Spatio-temporal variation of bacterioplankton community structure in the Pearl River: impacts of artificial fishery habitat and physicochemical factors

**DOI:** 10.1186/s12862-022-01965-3

**Published:** 2022-02-03

**Authors:** Sheng Bi, Han Lai, Dingli Guo, Xuange Liu, Gongpei Wang, Xiaoli Chen, Shuang Liu, Huadong Yi, Yuqin Su, Guifeng Li

**Affiliations:** 1 State Key Laboratory of Biocontrol, Southern Marine Science and Engineering Guangdong Laboratory (Zhuhai) and Guangdong Provincial Key Laboratory for Aquatic Economic Animals, School of Life Sciences, Sun Yat-Sen University, Guangzhou, 510275 China; 2 School of Agriculture, Sun Yat-Sen University, Guangzhou, China; 3 Guangdong Provincial Engineering Technology Research Center for Healthy Breeding of Important Economic Fish, Guangzhou, 510006 China; 4 Zhongshan Ophthalmic Center, Sun Yat-Sen University, Guangzhou, 510006 China; 5 School of Life Sciences, Institute of Aquatic Economic Animals, Sun Yat-Sen University, No. 132, East Outer Ring Road, Guangzhou, 510006 China

**Keywords:** Bacterioplankton community, Bacterial diversity, Microbial ecology, Artificial fishery habitat, Pearl River

## Abstract

**Background:**

Artificial fishery habitat has been widely used in fishery resource protection and water habitat restoration. Although the bacterioplankton plays an important ecological role in fisheries ecosystems, the effect of artificial fishery habitat on bacterioplankton is not clear. In this study, high-throughput sequencing based on the 16S rRNA gene was carried out to study the characteristics of bacterioplankton community structure in artificial fishery habitat and to determine the principal environmental factors that shaped the composition, structure and function of bacterioplankton communities in an unfed aquaculture system.

**Results:**

The results indicated that the most dominant phyla were Proteobacteria (Alphaproteobacteria and Gammaproteobacteria), Actinobacteria, Cyanobacteria, and Bacteroidetes, which accounted for 28.61%, 28.37%, 19.79%, and 10.25% of the total abundance, respectively. The factors that cause the differences in bacterioplankton community were mainly manifested in three aspects, including the diversity of the community, the role of artificial fishery habitat, and the change of environmental factors. The alpha diversity analysis showed that the diversity and richness index of the bacterioplankton communities were the highest in summer, which indicated that the seasonal variation characteristics had a great influence on it. The CCA analysis identified that the dissolved oxygen, temperature, and ammonium salt were the dominant environmental factors in an unfed aquaculture system. The LEfSe analysis founded 37 indicator species in artificial structure areas (AS group), only 9 kinds existing in the control areas of the open-water group (CW group). Meanwhile, the KEGG function prediction analysis showed that the genes which were related to metabolism in group AS were significantly enhanced.

**Conclusions:**

This study can provide reference value for the effect of artificial habitat on bacterioplankton community and provide fundamental information for the follow-up study of ecological benefits of artificial fishery habitat. It may be contributed to apply artificial fishery habitat in more rivers.

**Supplementary Information:**

The online version contains supplementary material available at 10.1186/s12862-022-01965-3.

## Background

In recent decades, with the rapid growth of population and the construction of dams in the Pearl River Basin, the water environment has inevitably deteriorated to a certain extent. Water eutrophication and habitat fragmentation have become the main problems affecting the development of river fisheries and the dynamics of planktonic microorganisms. In this context, it is necessary to construct the artificial fishery habitat for the restoration of aquatic ecology, and which has become an ideal carrier for studying the dynamic characteristics of the bacterioplankton community under the influence of natural and human forces.

The bacterioplankton community is one of the vital parts of the aquatic ecosystem. It has played many key roles in the process of biogeochemical circulation, including ammonia oxidation, degradation, adsorption, fixation of carbon dioxide and nitrogen [1–5].It is an important goal of microbial ecology to study that which habitat or environmental factors can affect the bacterioplankton community structure, and revealing the spatio-temporal variation of the bacterioplankton community is helpful to clarify the variation process and functional maintenance of microorganisms [6, 7]. In the aquatic system, the bacterioplankton communities are highly dynamic [8–10], because that their composition, diversity, and function may be influenced by environmental changes [11, 12]. Previous studies had shown that spatial and temporal variations in the community structure of the bacterioplankton were affected by many environmental factors, such as temperature [13], dissolved oxygen levels [14], pH [15], nutrient [16], organic matter [17] and salinity [18]. It had been manifested that the bacterioplankton community composition followed a pattern of annual variation and showed a predictable temporal pattern in a different environment [19, 20]. The seasonal variation of bacterioplankton community structure in an urban disturbed river was primarily regulated by temperature [21]. At the same time, some scholars had shown that the spatial variation which was from the water surface to bottom in the bacterioplankton community could be greater than that of seasonal change [22].

Artificial fishery habitat can significantly increase species diversity and spatial heterogeneity and has an obvious aggregation effect on fish [23, 24]. As such, when the artificial habitat had performed these functions, they were likely to inevitably affect the bacterioplankton community, including the density of food increased, the biodiversity of fish increased, and the physical and chemical factors changed [25, 26]. Although some studies on the dynamics of the bacterioplankton community in the Pearl River had been reported [27–30], there were relatively few studies on the spatio-temporal characteristics and effects of the bacterioplankton community in the artificial fishery habitat of Pearl River. This study aimed to characterize and compare the bacterioplankton communities of temporal and spatial change patterns in artificial fishery habitats, using 16S rRNA high-throughput sequencing. These results could enhance our understanding about the composition, diversity and predictive functions of bacterioplankton communities in artificial fishery habitats.

## Results

### Fish assemblages and environmental variables

During the experiment, 3825 individual fish of 36 fish species, representing four orders and nine families, were recorded in the natural and artificial fishery habitats. Among them, 2362 individual fish of 34 species were found at AS group and the total weight of the AS group was 48,160 g, while 1,463 individual fish of 29 species were found at the CW group and the total weight of the CW group was 24,730 g. From the number of fish, the AS group was 1.61 times of the CW group whereas from the quality of fish, the AS group was 1.95 times of the CW group. These results indicated that the spatial heterogeneity of artificial fishery habitat played an important function in enriching and stabilizing on fish assemblages.

The corresponding physicochemical factors of 192 samples were simultaneously monitored (Additional file 1: Table S1–S4). The fluctuation of water temperatures (20.2–27.3 °), total dissolved solids (139–196 mg/L), chlorophyll-a (0.1–19.22 μg/L), ammonium salts (1.23–3.13 mg/L) and dissolved oxygen (5.89–8.10 mg/L) were large. The pH-values ranged from 7.25 to 8.48. The range of baro (989–1009 mb), salinity (0.07–0.1 ppt) and water depth (0–6.15 m) fluctuation were small.

### Spatio-temporal variation in bacterioplankton community composition, structure and diversity

**Fig. 1:**
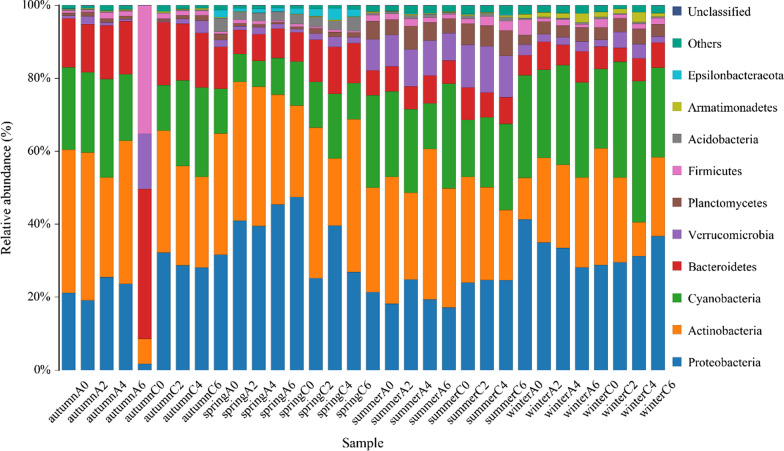
The composition of the bacterioplankton community at phylum levels in all samples. Only the top 10 taxa with the largest average relative abundance are listed

By HiSeq sequencing, the16S rRNA gene amplification products of all samples were detected and a total of 2,415,151 high-quality sequences were obtained. The number of sequences with each sample ranged from 33,199 to 70,515, with an average of 52,679 sequences per sample. According to the 97% similarity threshold, the sequences were divided into different operational taxonomic units (OTUs). In order to compare, the abundance of OTUs was standardized to 33,199 sequences. Of the classifiable sequences, 32 phyla were identified. The most dominant community members, Proteobacteria including Alphaproteobacteria and Gammaproteobacteria, Actinobacteria, Cyanobacteria, Bacteroidetes, Verrucomicrobia, Planctomycetes, Firmicutes, Actinobacteria, Armatimonadetes and Epsilonbacteraeota accounted for 28.61, 28.37, 19.79, 10.25, 4.34, 2.71, 2.39, 1.06, 0.55, and 0.48% of total abundance, respectively (Fig. 1).

**Fig. 2:**
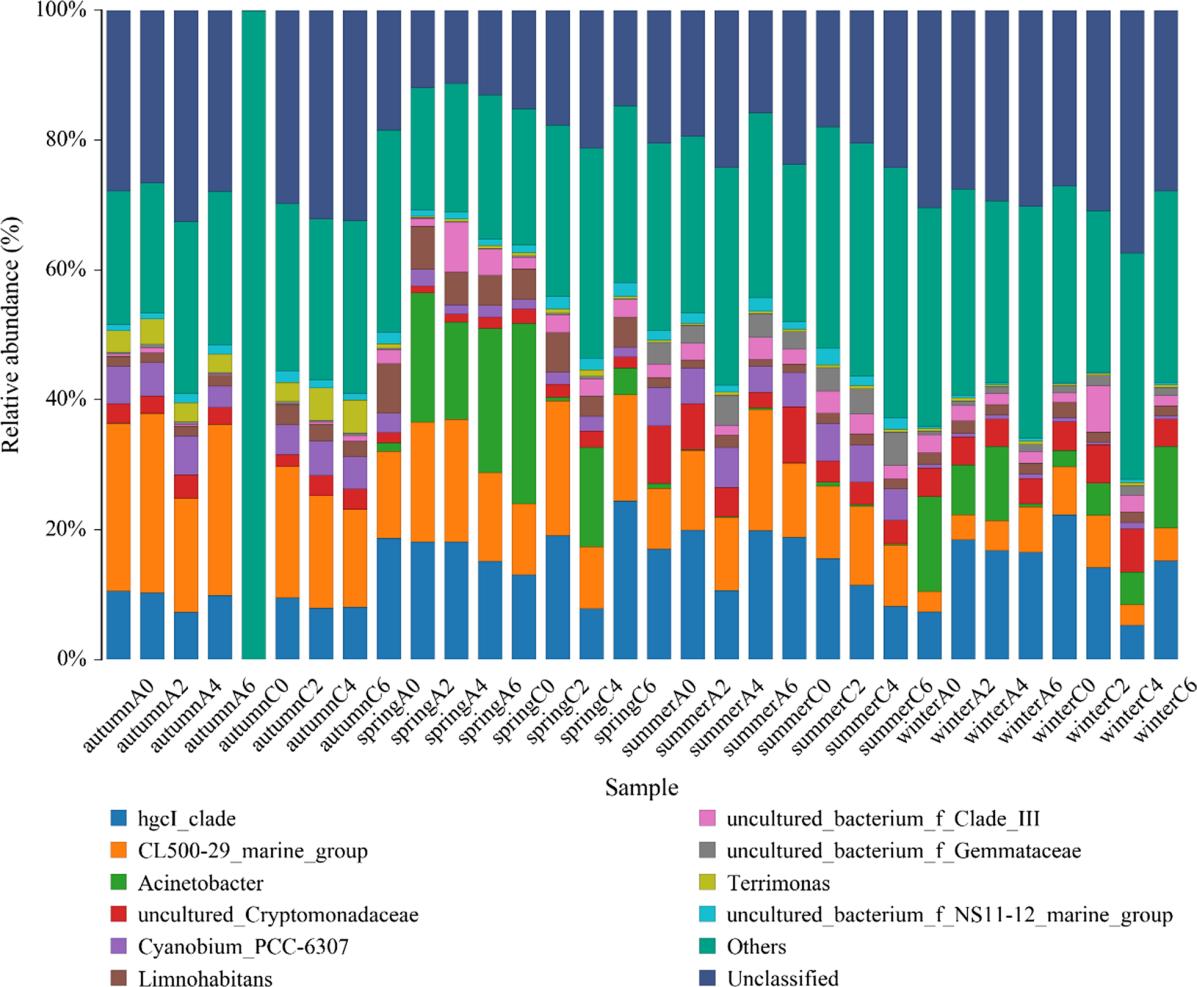
The composition of the bacterioplankton community at genus levels in all samples. Only the top 10 taxa with the largest average relative abundance are listed

At the genus level, 354 genera were identified. The most abundant groups were *hgcI_clade*, *CL50029_marine_group*, *Acinetobacter*, *Cyanobium_PCC6307*, *Limnohabitans* and *Terrimonas*, which accounted for 13.6%, 12.8%, 5.24%, 3.90%, 2.60%, and 1.14% of the total bacterioplankton community, respectively (Fig. 2).

**Fig. 3:**
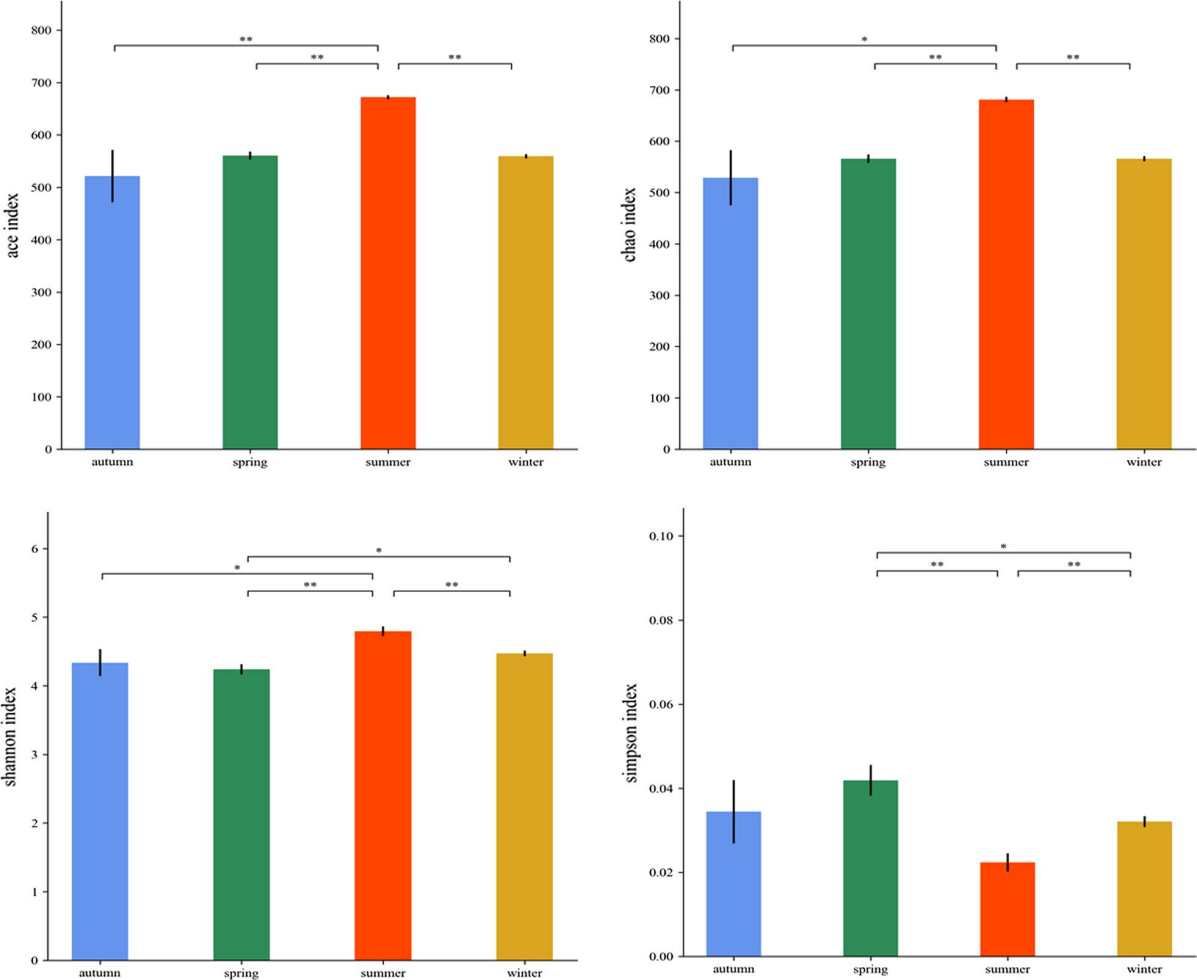
Comparison of alpha diversities, including Abundance-based Coverage Estimator (ACE), Chao-1, and Shannon and Simpson indices among the four seasons

The sparse curves of all samples in the AS group and CW group had reached the plateau stage, indicating that the quantitative analysis of the sequence fully represents the bacterioplankton diversity (Additional file 1: Fig. S1). In terms of diversity indexes, the average values of ACE, Chao1, Shannon and Simpson were 578, 585, 4.46 and 0.03, respectively. The ANOVA test followed by post hoc comparison showed that the species richness indexes (Chao1 and ACE) in summer was higher than that in other seasons, and the difference was significant (*P* < 0.05). The seasonal diversity indexes (Shannon and Simpson) showed significant temporal variability (Fig. 3). In summer, the Shannon index was the highest, and the Simpson index was the lowest. Besides, there was no significant difference in the Simpson index between autumn and other seasons.

The Shannon, Chao1 and ACE indexes of the AS group were always higher than those of the CW group, but the result of the Simpson index was in reverse (Table 1). The ANOVA test showed that there were no significant differences in the species richness index (Chao1 and ACE) between AS group and CW group, but there were significant differences in diversity indexes (Shannon and Simpson) between the two groups (Table. 1).

**Table 1:** The alpha-diversity analysis of the bacterioplankton community from AS group and CW group are shown

Diversity indexes	AS	CW
Shannon	6.335 ± 0.231^a^	5.051 ± 0.329^b^
Simpson	0.015 ± 0.002^a^	0.830 ± 0.08^b^
Chao-1	599 ± 31^a^	563 ± 20^a^
ACE	608 ± 34^a^	584 ± 18^a^

The means ± SD data of Table 1 in the same row with different letters differ significantly (*P* < 0.05)

**Fig. 4:**
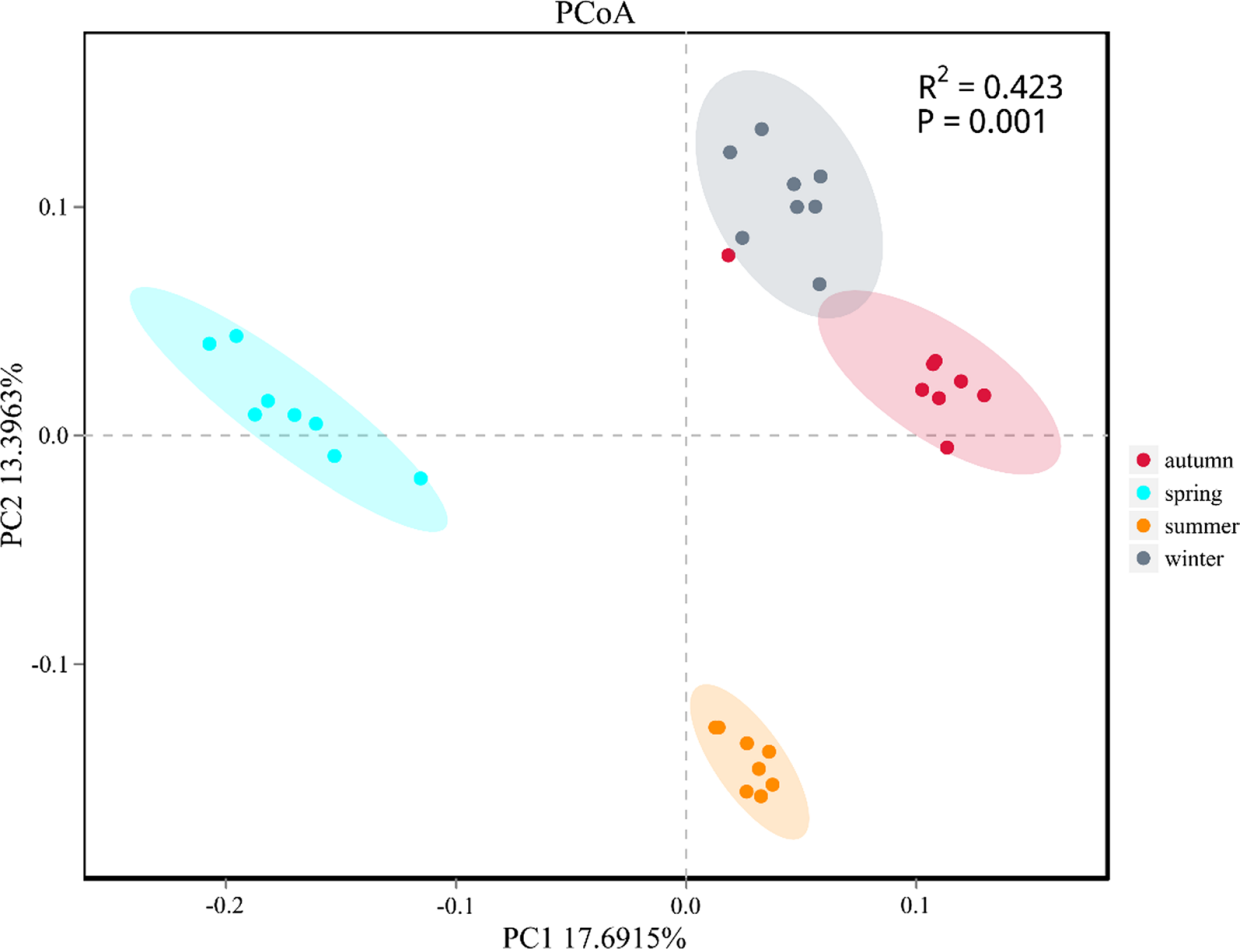
The results of the PCoA analysis among the four seasons

The spatial and temporal distribution of the bacterioplankton community was analyzed by the PCoA based on the Bray–Curtis dissimilarity index of samples. The results showed that the bacterioplankton community could be divided into four communities with different seasonal variations (Fig. 4, ANOSIM, P < 0.01). At the same time, according to the annual change of AS group and CW group, there was no obvious independent community between the two groups (Additional file 1: Fig. S2).

### Effects of artificial habitat and environmental variables on bacterioplankton communities

**Fig. 5:**
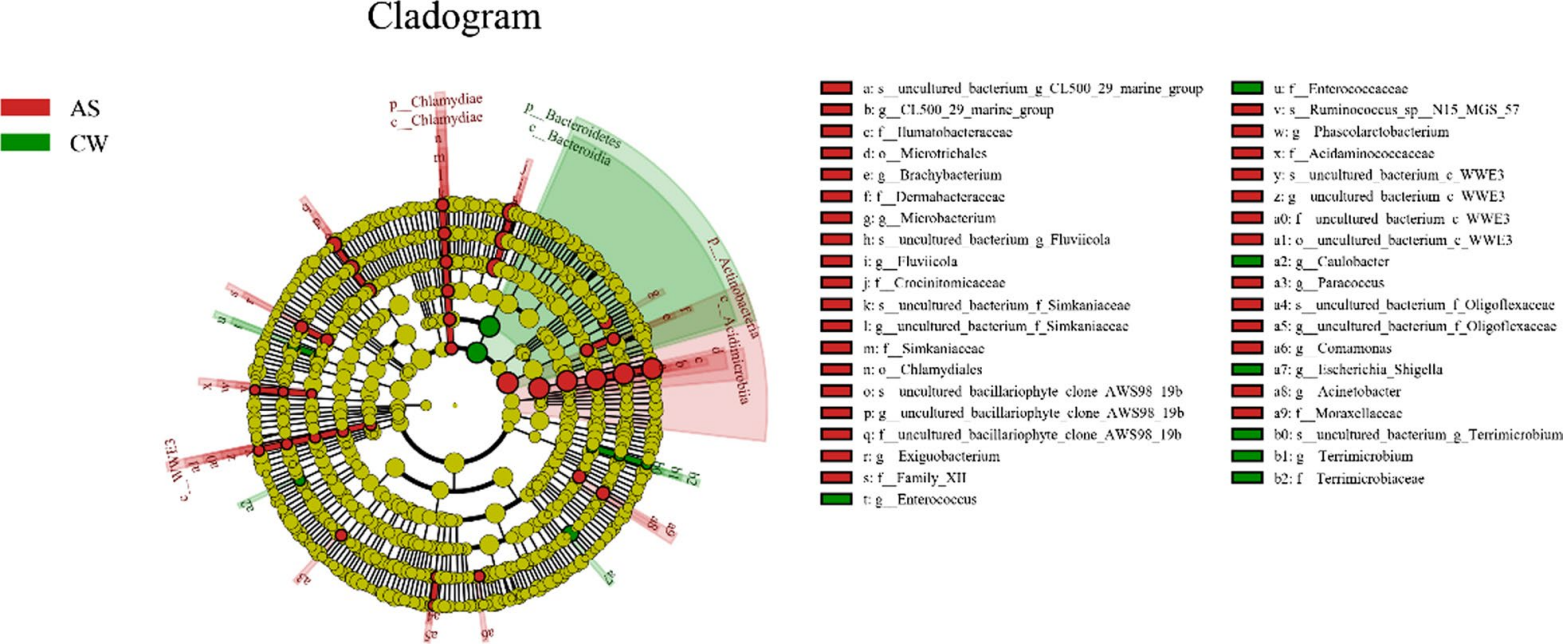
Linear effect size (LEfSe) analysis identified the most differentially abundant taxa (*P* < 0.05, LDA values > 2.5) between AS group and CW group. Differentially abundant taxa of each group are distinguished by different colors (red and green represented for AS and CW, and yellow for non-significant). Inside-out radiating circles represent taxonomic levels from phylum to genus

To study the effect of artificial fishery habitat (AS) on the bacterioplankton community, LEfSe analysis was used to identify the indicators with a great difference among different sites. It was observed that the dominant indicator species of bacterioplankton had changed significantly between the AS group and the CW group (Fig. 5 and Additional file 1: Fig. S3). The 37 indicators were influenced by AS group, whereas only 9 kinds were identified in the control area (*P* < 0.05, LDA > 2.5). The dominant taxa of the communities in the AS group were Actinobacteria, Ilumatobacteraceae, Microtrichales and Acidimicrobiia, and in CW group the dominant taxa were Bacteroidetes, Bacteroidia and Escherichia Shigella under the condition of LDA > 3 (Fig. 5 and Additional file 1: Fig. S3).

**Fig. 6:**
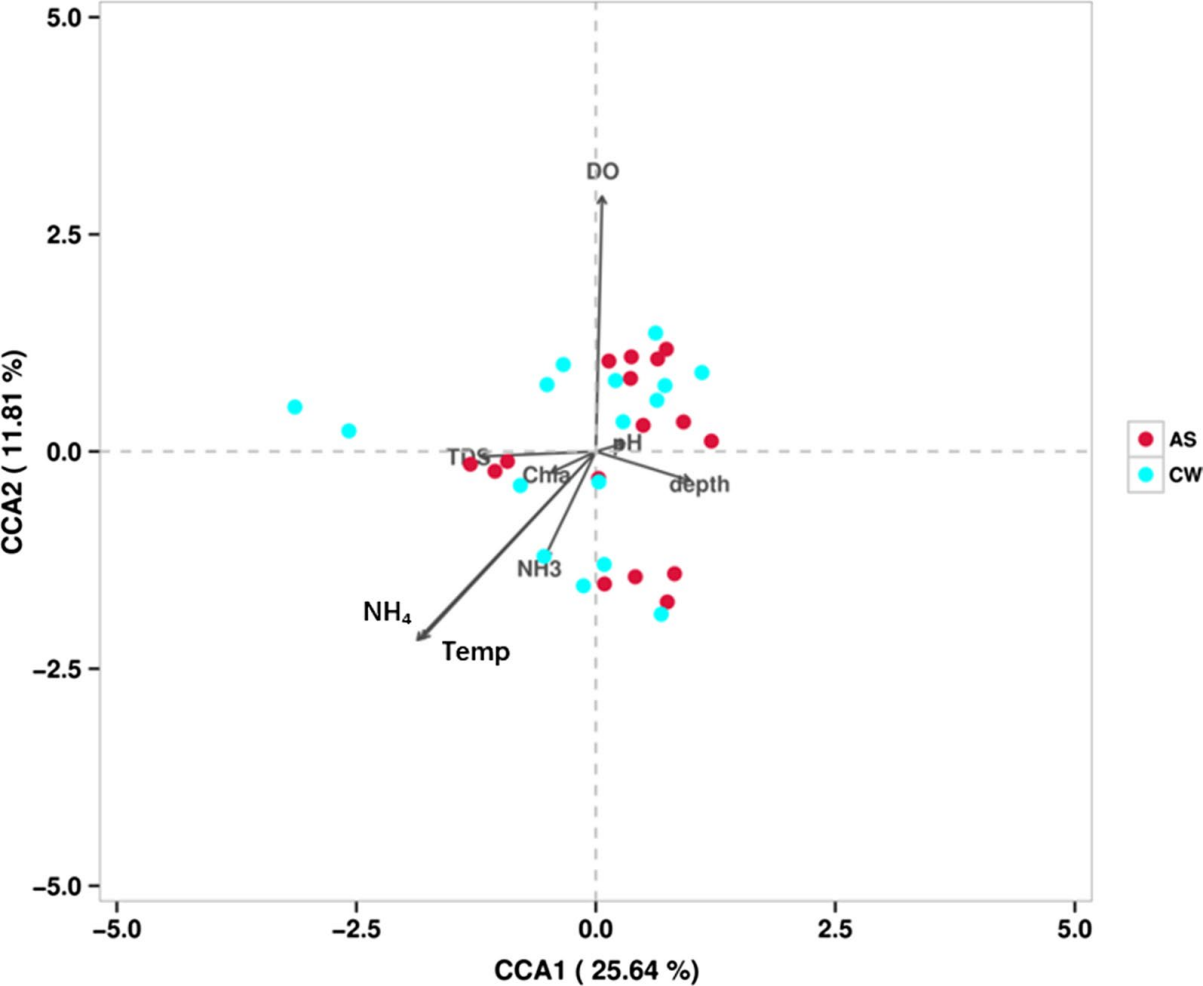
The CCA (canonical correspondence analysis) results of environmental variables between group AS and CW

Based on those analyses, the CCA analysis (Canonical Correlation Analysis) was carried out to study the relationship between the bacterioplankton community and environmental factors (Fig. 6). The results showed that temperature (r^2^ = 0.951, *P* = 0.001), dissolved oxygen (r^2^ = 0.892, *P* = 0.003) and ammonium salt (r^2^ = 0.942, *P* = 0.001) had significant correlations with the bacterioplankton community. There was no significant correlation between other environmental factors and bacterioplankton community (*P* > 0.05). The results showed that dissolved oxygen was probably the most important environmental factor (i.e., the longest arrow), which was the high correlation with the bacterioplankton community.

### Potential functional consequences

**Fig. 7:**
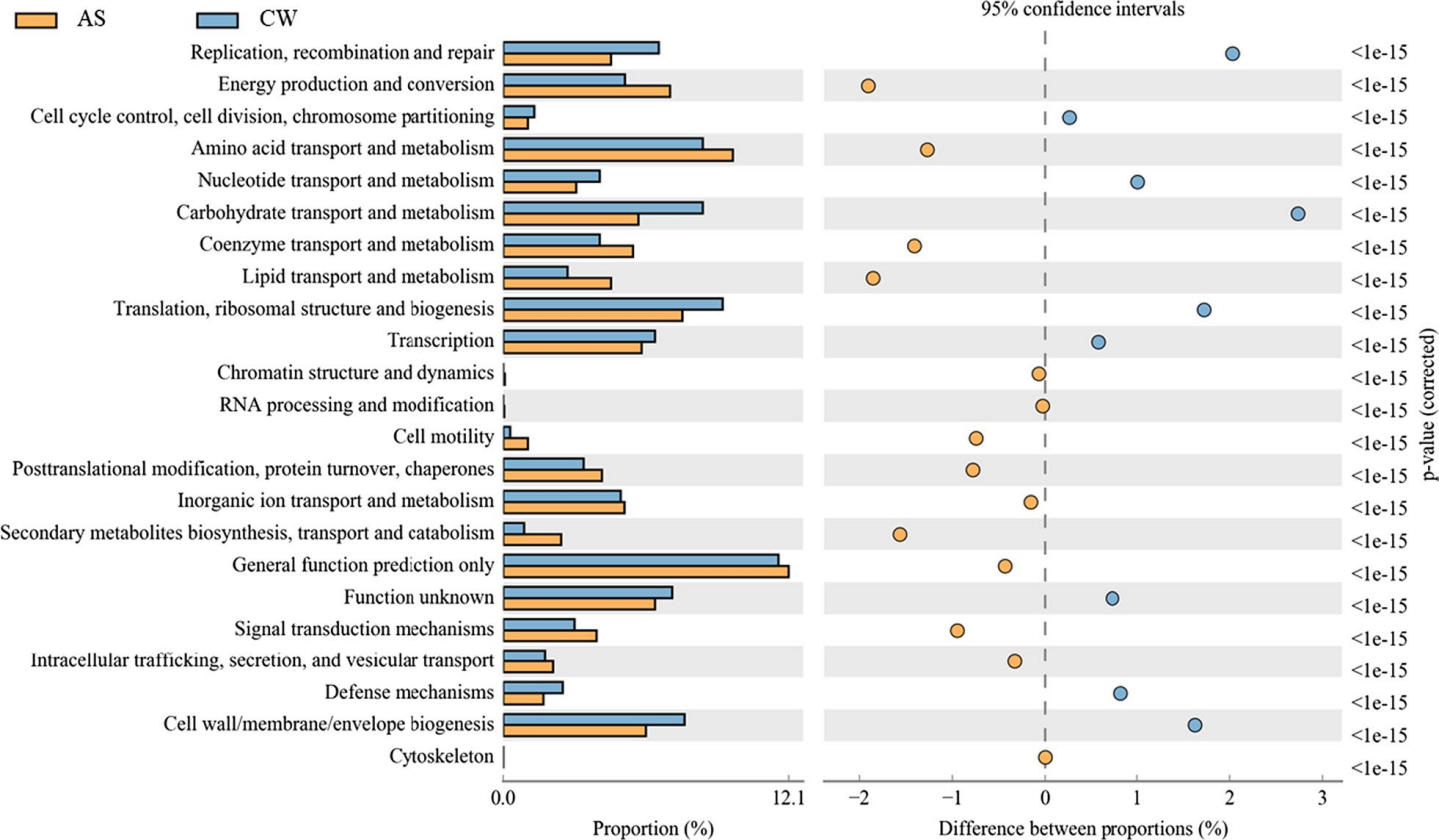
The KEGG metabolic pathways in AS and CW groups

The PICRUSt was used to predict the function of 16S rRNA gene amplicons to analyze the function of the bacterioplankton community. The results of difference analysis by KEGG metabolic pathway indicated that some predictive pathways were significantly enriched in the AS group (95% confidence intervals, *P* < 0.05), compared with the CW group. Especially, these genes were associated with metabolism processing (e.g., amino acid transport and metabolism, coenzyme transport and metabolism, energy production and conversion, etc.) (Fig. 7). At the same time, the enhancement of metabolic function usually means the increase in oxygen consumption. Therefore, referring to the CCA correlation analysis of environmental factors in Fig. 6, we can confirm that dissolved oxygen is the most significant environmental factor in artificial fishery habitat, which affects the bacterioplankton communities.

## Discussion

This study aimed to characterize and compare the bacterioplankton communities of temporal and spatial change patterns in artificial fishery habitats, and to clarify the bacterioplankton communities difference among diversity, composition, and predictive function in artificial fishery habitat.

It had been reported that both river and marine environment, the bacterioplankton community structure in aquatic organisms showed a certain spatial pattern, and the bacterioplankton community compositions also showed strong seasonal changes [31–34]. However, most studies had focused on the spatial or seasonal scales [35], and few studies had evaluated the effects of habitat effects of artificial fishery on planktonic microorganisms at the spatial and seasonal scales. At present, the researches about artificial habitats mainly have focused on fish reproduction and protection of larvae and juveniles [23, 36]. Besides the restoration of fishery resources, the artificial habitat could also form the spatial heterogeneity and flow field effect in the rivers [24], which will inevitably affect the bacterioplankton community structure. It was found that the Proteobacteria and Actinobacteria were the main planktonic bacteria in the Pearl River, accounting for more than half of the total (Fig. 1). This phenomenon is consistent with historical research [37–41]. In the carbon and nitrogen cycle of aquatic organisms, the Proteobacteria were the dominant microorganisms in the freshwater environments [42]. Besides, the Actinobacteria could adapt to various freshwater ecosystems because of their good plasticity [43]. The widespread distributions of these groups in aquatic ecosystems were consistent with their ecological functions.

**Fig. 8:**
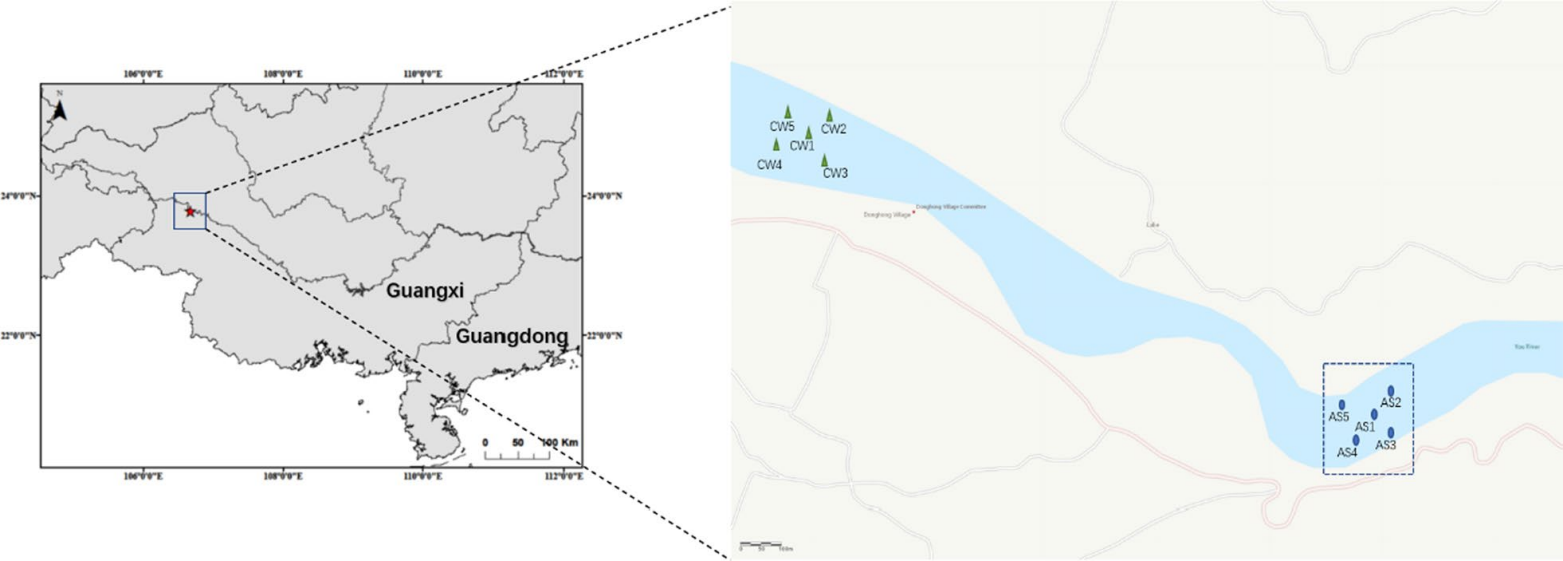
Study sites in Pearl River: artificial structure areas (AS) and control areas of the water (CW). The maps were made by ArcGIS 10.5 software

One of the common characteristics of the bacterioplankton community in various aquatic ecosystems seems to be the seasonal dynamics [41]. In the experimental station of Youjiang River (Fig. 8), we analyzed the seasonal variation characteristics of the bacterioplankton community in artificial habitat. According to the PCoA analysis, the bacterioplankton communities from all sampling sites showed significant seasonal changes, which were divided into four groups: spring, summer, autumn and winter. At the same time, the variation ranges in summer were the most concentrated (Fig. 4). On the other hand, the seasonal variation of artificial habitat and control area almost completely overlapped (Additional file 1: Fig. S2), which also indicated that the response of bacterioplankton communities to seasonal changes were more significant, and could almost cover the influence of other factors. In inland rivers, there are different microbial communities in different seasons, which are usually caused by major seasonal events such as temperature, primary productivity and hydraulic flood discharge [44, 45]. Comparing the alpha diversities (Chao-1, ACE, Shannon and Simpson) of the bacterioplankton community could find that the three groups of indexes showed a trend of first increasing and then decreasing with the seasonal change, except for the Simpson index (Fig. 3). Especially in summer, the three groups indexes reached their maximum values (*P* < 0.05), while the Simpson index reached the minimum value (*P* < 0.05). Similar to PCoA analysis, both the diversity indexes and richness indexes of the bacterioplankton community had increased significantly in summer. This result mean that the temperature may be a key environmental factor. Some studies suggested that the bacterioplankton had important ecological functions, such as regenerating nutrients, decomposing organic matter, and participating in the basis of the microbial food web, which were regulated by the temperature [46, 47].

In the aquatic ecosystem, the bacterioplankton community was relatively sensitive to environmental disturbance. For example, the composition of bacterioplankton community usually could be affected by the temperature change, habitat characteristics, dissolved oxygen change and other factors [5, 48]. Spatial variability can reflect the changes of key environmental factors, which affect the growth and survival of microorganisms [9]. On this basis, artificial fishery habitat, as a special structure, that could not only change the water environment but also increase spatial heterogeneity. According to the CCA analysis, it could be easily found that temperature, dissolved oxygen and ammonium salt were the most important factors, and dissolved oxygen was the most relevant (Fig. 6). First of all, the effect of temperature change has been confirmed due to seasonal changes. Secondly, as far as dissolved oxygen is concerned, the complex structure of artificial fishery habitat will change the flow velocity and intercept some phytoplankton to increase the intensity of photosynthesis [49]. While the fish gathered in artificial fishery habitat, the dissolved oxygen could be fluctuating. At the same time, when the columnar structure was submerged in the water from top to bottom, a part of upwelling would be formed [49], which will increase the disturbance of the water body, and help the gas exchange between the upper and lower water bodies and achieve the function of equal distribution. The submersion of columnar structure would enrich some nutrients in a short term. The above aspects work together to keep the dissolved oxygen energy relatively stable and become the key factor. Finally, as for the discussion of ammonium salt, it was found that there were some residents gathered along the control area (group CW), according to the field investigation, whose discharge of the domestic water and production water was not controlled reasonably, so it would inevitably cause pollution to the waters near the Youjiang River to a certain extent. This pollution may be reflected in the correlation between the bacterioplankton community and ammonium concentration.

The dynamic change of the bacterioplankton community was related to its functional characteristics. According to the LEfSe analysis (Fig. 5 and Additional file 1: Fig. S3), there were great differences about the identified indicator species between AS group (37 indicators) and CW group (9 indicators). This result mean that the indicator species in the artificial fishery habitat were more extensive and stable, and responded more quickly to environmental changes. On this basis, the KEGG function prediction analysis showed that the metabolic pathways of the bacterioplankton community (such as amino acid transport and metabolism, coenzyme transport and metabolism, energy production and conversion, etc.) in artificial fishery habitat were significantly increased, compared with the control group (Fig. 7). From this point of view, the enhancement of metabolic function often increased the oxygen consumption demand [50], which further verified the conclusion of CCA analysis that dissolved oxygen was the key influencing factor. At the same time, it also reflected the potential regulation effect of artificial fishery habitat to dissolved oxygen.

## Conclusions

This study represented an attempt to study the bacterioplankton community in artificial fishery habitats and analyzed the response of the bacterioplankton community structure to different seasons and water depth in an unfed aquaculture system. We revealed that, in terms of temporal scale, seasonal variation had the most significant effect on the bacterioplankton community structure, and the diversity indexes and richness indexes reached the maximum values in summer. From the spatial scale, the water ecological protection effect of artificial fishery habitat was better than that of the control group. The spatial heterogeneity of the artificial habitat made the temperature, dissolved oxygen and water depth become the key factors affecting the bacterioplankton community. From the perspective of temporal and spatial patterns, the construction of artificial fishery habitats were conducive to the stability and enrichment of the bacterioplankton community structure, to gather more fish, which could be better to play its ecological benefits.

## Methods

### Study sites and artificial fishery habitats

The artificial fishery habitats located between the cascade water control projects in the Youjiang river section of the Pearl River Basin, and the blue dotted box indicated the areas where the artificial fishery habitats were placed. An unfed aquaculture program had been implemented in this river to reduce the eutrophication of the river (Fig. 8).

**Fig. 9:**
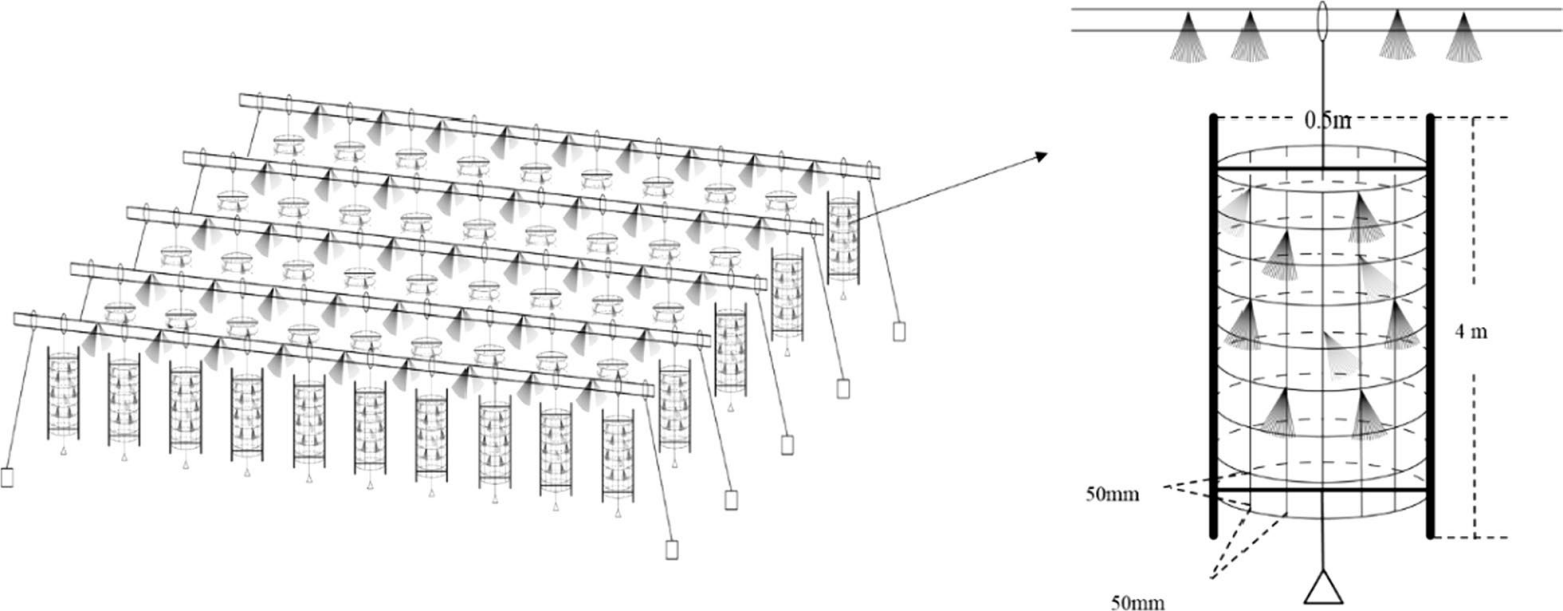
The schematic diagram of artificial habitat. This type of artificial fishery habitat was only placed in AS group, and CW group was not placed as a blank area [51]

Two types of habitats were selected, the artificial structure areas (AS) and nearby control areas of the water (CW), and the five sampling duplicates were randomly selected. The Fig. 8 showed the location of ten sampling sites in the AS (AS1–AS5) and CW areas (CW1–CW5) in Pearl River. Artificial fishery habitats were consisted of two parts: floating raft and cylindrical frame structure. The floating raft was formed by series binding of bamboos with a diameter of more than 20 cm, which provided buoyancy and stabilized the artificial fishery habitats. The cylindrical frame structure was made of tough bamboo skin with a grid structure. The radius of the frame structure was about 0.5 m, the height was about 8 m which could be adjusted according to the actual needs, and the grid size was about 5 × 5 cm. Palm skin was laid on floating row and cylindrical frame structure to provide substrate for algae growth and fish spawning (Fig. 9) [25, 51]. An anchor was attached to the bottom of the cylindrical frame structure to make the cylindrical frame structure vertical in water. On this basis, more than 1000 structural units were constructed and laid on the experimental sites in an orderly way, covering an area of about 2000 square meters, namely, the artificial fishery habitats. The artificial habitat had been applied into the experimental sites of Youjiang River in December 2015. In terms of time scale, the artificial habitat has been put into use for a relatively long time, and a relatively stable and mature ecosystem has been formed. Four water layers of AS group and CW group were selected to collect and analyze water samples.

### Sample collection and physicochemical analysis

During the summer (June) of 2018 to spring (March) of 2020, 192 water samples in the experimental area and the control area of the artificial structures were collected in four different depths (0, 2, 4 and 6 m) in 8 quarters (Fig. 1). Each of the two groups had 5 randomly repeated sampling points which include AS1-AS5 and CW1-CW5. For each sample, 1.0 L of water was taken from the fixed depth of the water layer using a sterile bottle, and the sterile bottle containing the water sample was immediately placed on the ice [52], and then filtered through 0.22 μM the porous polycarbonate membrane (Millipore, MA, USA) by Shimadzu vacuum membrane pump (VP–10L). The samples were stored in liquid nitrogen until DNA was extracted. Temperature, pH, DO, Chlorophyll-a and total dissolved solids were measured by Macro-900 (Palintest, Germany) handheld multi-parameter field instrument (Additional file 1: Table S5). A total of 192 water samples were collected, and about 1000 ml of each water sample was used to measure the physicochemical factors by Macro-900. All fish were sampled using multimesh gillnets that were 18 m long, 1.5 m height with mesh sizes between 6.25 and 60 mm of the following order: 45, 20, 6.25, 10, 55, 40, 12.5, 25, 15, 60, 35, and 30 mm [53]. All fish were then anesthetized using the 40 mg/L of tricaine methanesulfonate (MS-222) for subsequent sampling.

### DNA extraction, PCR amplification, and 16S rRNA sequencing

DNA was extracted from all samples using a Bacterial DNA kit (MN NucleoSpin^®^ 96 Soi, Germany) following the manufacturer’s instructions. The concentration and purity of genomic DNA were detected using a Nanodrop 2000c Spectrophotometer (Thermo Scientific, DE, USA). In order to carry out 16S rRNA gene amplification analysis, we amplified the v3-v4 hypervariable region of the 16S rRNA gene using 338F (ACTCCTACGGGAGGCAGCA) and 806R (GGACTACHVGGGTWTCTAAT) primers [12, 40]. PCR reactions were carried out using Phusion High-Fidelity PCR Master Mix (New England Biolabs, UK). The amplification followed a set procedure: denaturation at 94 °C (5 min), then 35 cycles at 94 °C (30 s), 53 °C (30 s), and 72 °C (30 s), with the final elongation at 72 °C (10 min). and the PCR products were pooled at equimolar concentrations. The libraries were sequenced with the Illumina HiSeq2500 system (Illumina, USA) according to the standard protocols at Biomarker Technologies Co. Ltd (Beijing, China). The 16S high-throughput sequencing raw data was uploaded in the Dryad Sequence Read Archive, which is the download link https://doi.org/10.5061/dryad.tqjq2bvxm.

### Processing of sequencing data

The raw reads which were generated from the HiSeq2500 system were merged using FLASH (Version 1.2.11) [54]. To improve the quality of sequencing data, the quality of the spliced sequences was filtered by Trimmmatic (version 0.3.3) [55], and simultaneously chimeras were removed by UCHIME (version 8.1) [56] to obtain high-quality 16S sequences. The effective tags were clustered into operational taxonomic units (OTUs) of 97% similarity threshold using the UPARSE (version 10.0) pipeline [57]. The representative sequence in each cluster from the tag sequence with the highest abundance was selected, and then the taxonomic information of groups were annotated for each representative sequence by the naive Bayesian model with the Ribosomal Database Project (RDP) classifier of 50% threshold [58] which were based on the SILVA (http://www.arb-silva.de) database [59]. PyNAST (version 1.2.2) was used for multiple sequence comparisons to determine the phylogenetic relationships of dominant species in different OTUs and different samples [60]. Chao-1 (richness estimator), Simpson (diversity index) and Shannon–wiener (diversity index) estimates were calculated based on the observed species, and the analyses were conducted in Mothur (Version 1.3.0) [61].

### Statistical analysis

Almost all statistical analyses were based on the use of R statistical software, using a variety of packages to achieve. The alpha diversity indices were calculated by Mothur, which were including Abundance-based Coverage Estimator (ACE), Chao1, Simpson, and Shannon indices. ANOVA test was used to compare the alpha diversity indices of bacterioplankton communities; *P* < 0.05 indicated the significant difference. Principal coordinates analysis (PCoA) was used to analyze the spatial and temporal distribution of the bacterioplankton community. To study the relationships between bacterioplankton communities and environmental factors, canonical correspondence analysis (CCA) was conducted using CANOCO 5.0 software [62].

The LEfSe analysis was performed using the online tool (http://huttenhower.sph.harvard.edu/galaxy/root?tool_id=lefse_upload) to find the indicator species that had significant differences between groups. Before the final metagenome prediction, OTUs were normalized by dividing the abundance by the known copy number abundance of the 16S rRNA gene. Functional changes in the bacterioplankton communities between experimental and control areas were predicted by using Phylogenetic Investigation of Communities by Reconstruction of Unobserved States (PICRUSt) [63]. We reconstructed the metagenome functional genes by rarefied 16S rRNA copy numbers, which were further classified via Kyoto Encyclopedia of Genes and Genomes (KEGG) categories at levels 1, 2, and 3 in both artificial habitats and control areas [64]. Additionally, Welch’s t-test was used to compare and identify the functional genes which were the most differential abundance between the two groups of samples.

## Supplementary Information

**Additional file 1: Table S1.** The environmental factors of the group AS and CW in autumn. **Table S2.** The environmental factors of the group AS and CW in winter. **Table S3.** The environmental factors of the group AS and CW in spring. **Table S4.** The environmental factors of the group AS and CW in summer. **Table S5.** The accuracy of the Macro-900 hand-held measurements. **Figure S1.** Rarefaction curves of the AS and CW group. **Figure S2.** PCoA plot of the Bray-Curtis distances between group AS and CW. **Figure S3.** Linear discrimination analysis (LDA) in relative values of all kinds.

## Data Availability

The data presented in this study are available on request from the corresponding author.
