# Supplementary material for: Spatio-temporal variation of bacterioplankton community structure in the Pearl River: impacts of artificial fishery habitat and physicochemical factors
Source: BMC Ecol Evol. 2022 Feb 3;22:10. doi: 10.1186/s12862-022-01965-3 (PMC8812236; doi:10.1186/s12862-022-01965-3)
Supplement: Supplementary file 1 — Additional file 1: Table S1. The environmental factors of the group AS and CW in autumn. Table S2. The environmental factors of the group AS and CW in winter. Table S3. The environmental factors of the group AS and CW in spring. Table S4. The environmental factors of the group AS and CW in summer. Table S5. The accuracy of the Macro-900 hand-held measurements. Figure S1. Rarefaction curves of the AS and CW group. Figure S2. PCoA plot of the Bray-Curtis distances between group AS and CW. Figure S3. Linear discrimination analysis (LDA) in relative values of all kinds. [file 12862_2022_1965_MOESM1_ESM.docx]

Table. S1 The environmental factors of the group AS and CW in autumn

| Autumn | Temp (℃) | Baro (mb) | Depth (m) | pH | DO (% Sat) | DO (mg/L) | TDS (mg/L) | SAL (ppt) | NH3 (mg/L) | Chlorophyll (ug/L) |
| --- | --- | --- | --- | --- | --- | --- | --- | --- | --- | --- |
| AS0 | 27.0 | 990 | 0.27 | 7.50 | 93.1 | 7.22 | 167 | 0.08 | 0.30 | 8.96 |
| AS2 | 26.8 | 990 | 2.25 | 7.70 | 93.4 | 7.27 | 169 | 0.08 | 0.24 | 3.40 |
| AS4 | 26.7 | 990 | 4.09 | 8.08 | 91.3 | 7.12 | 176 | 0.09 | 0.21 | 0.60 |
| AS6 | 26.1 | 990 | 6.13 | 8.06 | 88.0 | 6.94 | 183 | 0.09 | 0.18 | 0.20 |
| CW0 | 27.3 | 990 | 0.30 | 7.90 | 97.2 | 7.53 | 185 | 0.09 | 0.54 | 10.4 |
| CW2 | 27.0 | 990 | 2.05 | 8.10 | 95.8 | 7.43 | 187 | 0.09 | 0.32 | 5.72 |
| CW4 | 25.7 | 990 | 4.05 | 8.14 | 87.9 | 6.98 | 195 | 0.10 | 0.20 | 4.03 |
| CW6 | 25.7 | 990 | 6.09 | 8.07 | 88.0 | 6.99 | 196 | 0.10 | 0.17 | 0.81 |

Table. S2 The environmental factors of the group AS and CW in winter

| Winter | Temp (℃) | Baro (mb) | Depth (m) | pH | DO (% Sat) | DO (mg/L) | TDS (mg/L) | SAL (ppt) | NH3 (mg/L) | Chlorophyll (ug/L) |
| --- | --- | --- | --- | --- | --- | --- | --- | --- | --- | --- |
| AS0 | 22.8 | 1009 | 0.25 | 7.30 | 88.5 | 7.71 | 157 | 0.08 | 0.42 | 14.1 |
| AS2 | 21.9 | 1009 | 2.31 | 7.70 | 87.6 | 7.59 | 165 | 0.08 | 0.27 | 3.80 |
| AS4 | 21.5 | 1009 | 4.03 | 8.25 | 87.2 | 7.56 | 169 | 0.08 | 0.21 | 2.81 |
| AS6 | 21.2 | 1009 | 6.12 | 8.38 | 87.3 | 7.56 | 171 | 0.08 | 0.17 | 0.10 |
| CW0 | 21.9 | 1009 | 0.24 | 7.69 | 90.1 | 7.84 | 176 | 0.09 | 0.33 | 15.36 |
| CW2 | 21.4 | 1009 | 1.89 | 7.95 | 89.7 | 7.78 | 174 | 0.09 | 0.25 | 5.54 |
| CW4 | 21.3 | 1009 | 4.02 | 8.48 | 90.5 | 7.84 | 180 | 0.09 | 0.21 | 4.21 |
| CW6 | 21.2 | 1009 | 5.99 | 8.44 | 90.9 | 7.89 | 184 | 0.09 | 0.19 | 0.80 |

Table. S3 The environmental factors of the group AS and CW in spring

| Spring | Temp (℃) | Baro (mb) | Depth (m) | pH | DO (% Sat) | DO (mg/L) | TDS (mg/L) | SAL (ppt) | NH3 (mg/L) | Chlorophyll (ug/L) |
| --- | --- | --- | --- | --- | --- | --- | --- | --- | --- | --- |
| AS0 | 20.4 | 996 | 0.18 | 7.80 | 89.6 | 7.91 | 150 | 0.07 | 0.57 | 13.1 |
| AS2 | 20.3 | 996 | 2.12 | 8.12 | 90.1 | 7.97 | 154 | 0.08 | 0.40 | 4.58 |
| AS4 | 20.3 | 996 | 4.12 | 8.34 | 90.2 | 7.98 | 159 | 0.08 | 0.30 | 3.24 |
| AS6 | 20.2 | 996 | 6.15 | 8.48 | 89.9 | 7.96 | 164 | 0.08 | 0.17 | 1.02 |
| CW0 | 20.7 | 996 | 0.12 | 7.35 | 92.3 | 8.10 | 168 | 0.08 | 0.12 | 18.6 |
| CW2 | 20.6 | 996 | 1.96 | 7.98 | 91.1 | 8.01 | 168 | 0.08 | 0.11 | 3.25 |
| CW4 | 20.3 | 996 | 4.06 | 8.24 | 89.9 | 7.94 | 171 | 0.08 | 0.10 | 2.54 |
| CW6 | 20.2 | 996 | 5.98 | 8.32 | 89.5 | 7.93 | 174 | 0.09 | 0.08 | 0.98 |

Table. S4 The environmental factors of the group AS and CW in summer

| Summer | Temp (℃) | Baro (mb) | Depth (m) | pH | DO (% Sat) | DO (mg/L) | TDS (mg/L) | SAL (ppt) | NH3 (mg/L) | Chlorophyll (ug/L) |
| --- | --- | --- | --- | --- | --- | --- | --- | --- | --- | --- |
| AS0 | 25.8 | 989 | 0.15 | 7.43 | 75.8 | 6.01 | 139 | 0.08 | 0.4 | 14.62 |
| AS2 | 25.8 | 989 | 1.99 | 7.94 | 75.4 | 5.98 | 144 | 0.08 | 0.32 | 8.95 |
| AS4 | 25.6 | 989 | 3.95 | 8.26 | 76.2 | 6.04 | 173 | 0.08 | 0.27 | 5.99 |
| AS6 | 25.5 | 989 | 5.98 | 8.25 | 75.8 | 6.01 | 189 | 0.09 | 0.25 | 2.69 |
| CW0 | 25.9 | 989 | 0.10 | 7.25 | 75.2 | 5.95 | 161 | 0.08 | 1.77 | 19.22 |
| CW2 | 25.8 | 989 | 1.80 | 7.93 | 75.1 | 5.95 | 166 | 0.08 | 1.07 | 15.39 |
| CW4 | 25.8 | 989 | 3.93 | 8.22 | 74.4 | 5.9 | 176 | 0.09 | 0.69 | 8.32 |
| CW6 | 25.2 | 989 | 5.84 | 8.35 | 74.3 | 5.89 | 172 | 0.08 | 0.48 | 2.33 |

Table. S5 The accuracy of the Macro-900 hand-held measurements

| Macro-900 instrument | Temp (℃) | Depth (m) | pH | DO (% Sat) | DO (mg/L) | TDS (mg/L) | SAL (ppt) | NH3 (mg/L) | Chlorophyll (ug/L) |
| --- | --- | --- | --- | --- | --- | --- | --- | --- | --- |
| Resolution ratio | 0.1 | 0.01 | 0.01 | 0.1 | 0.01 | 1 | 0.01 | 0.01 | 0.01 |
| Accuracy | ±0.2 | ±0.01 | ±0.1 | ±0.1 | ±0.01 | ±1 | ±0.01 | ±0.01 | ±0.02 |


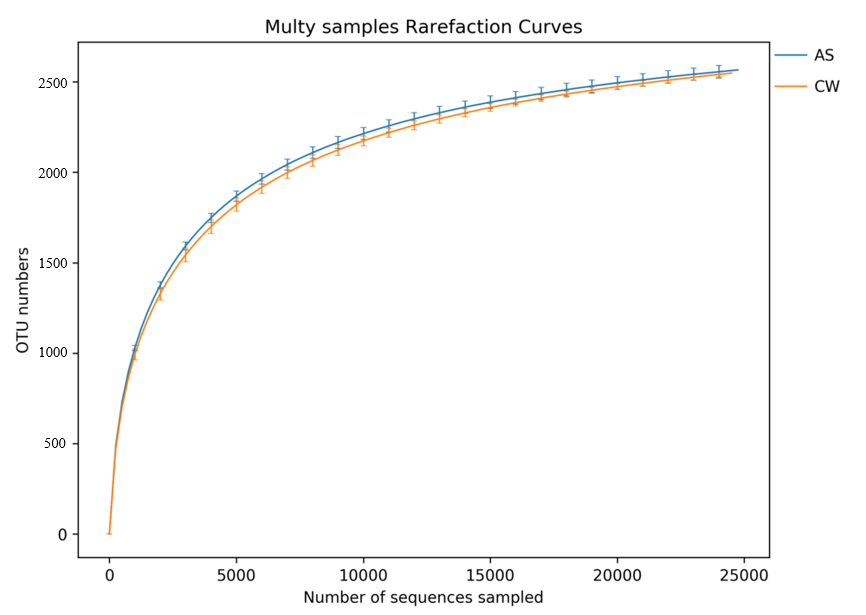


Figure. S1 Rarefaction curves of the AS and CW group


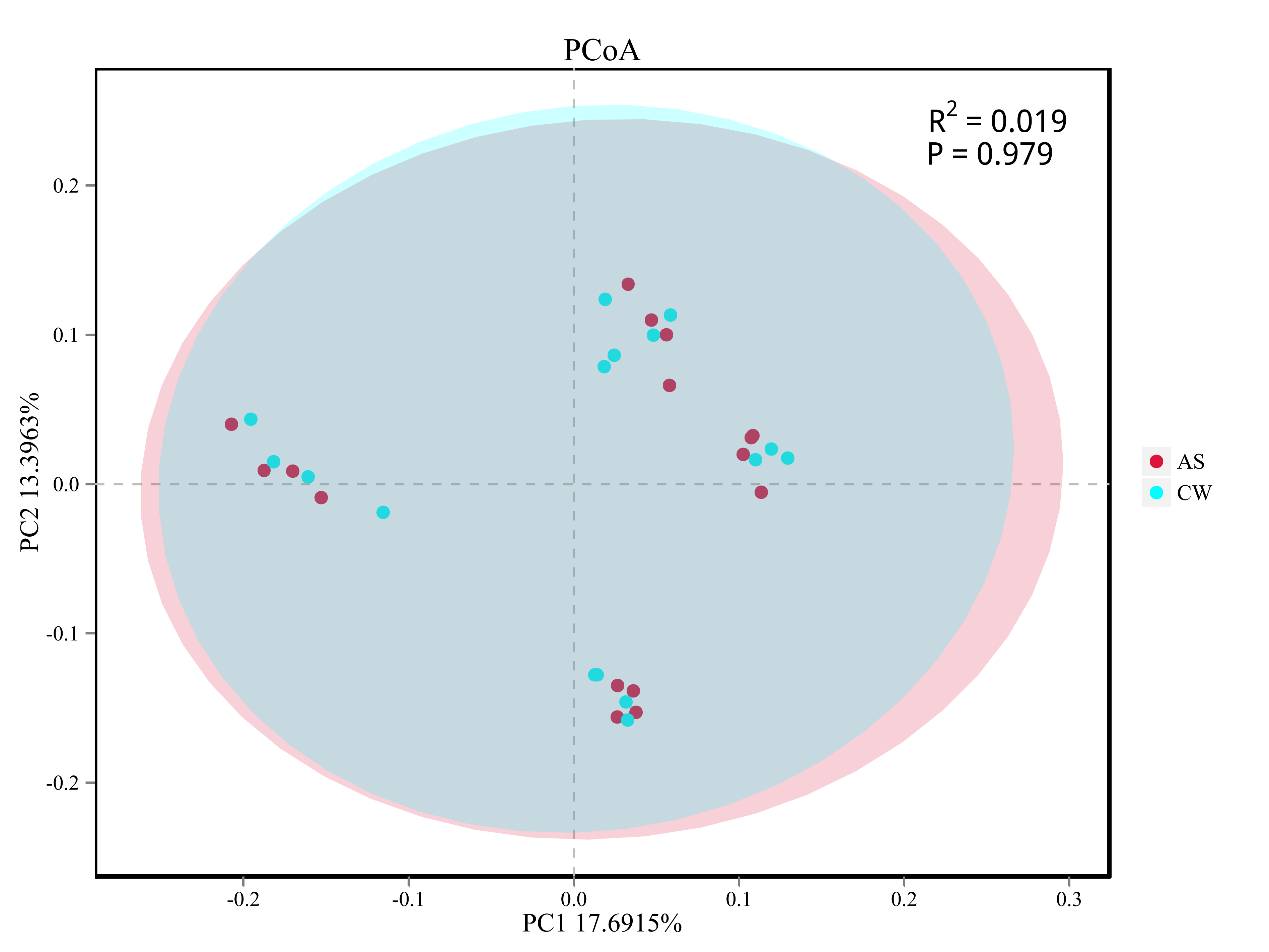


Figure. S2 PCoA plot of the Bray-Curtis distances between group AS and CW


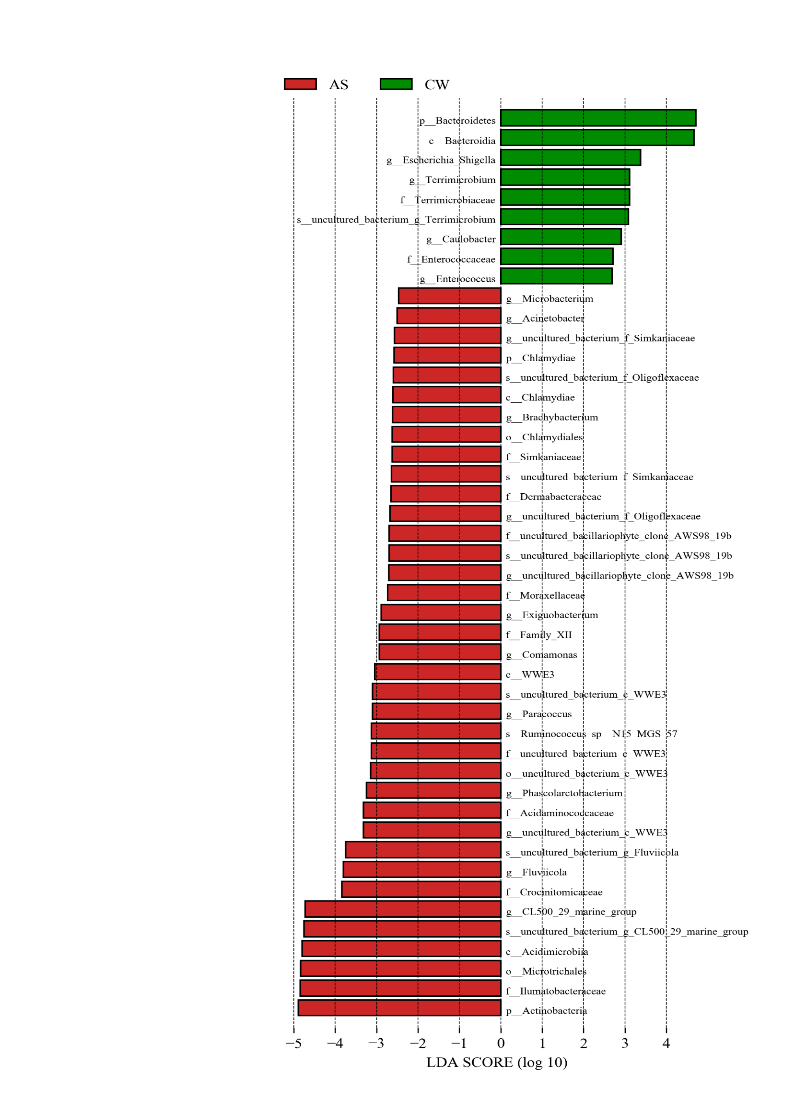


Figure. S3 Linear discrimination analysis (LDA) in relative values of all kinds
